# Mummification of the Dental Pulp

**Published:** 1900-01-15

**Authors:** 


### MUMMIFICATION OF THE DENTAL PULP.

Much of the success attained by modern methods of dental practice is directly the result of the degree of perfection that has been reached in the treatment of pulpless teeth. In fact, were it not for the possibility of saving that class of teeth in a condition of usefulness and comfort, not only would these be lost, but the elaborate systems of crown and bridge-work which owe their existence primarily to the salvability of pulpless teeth, would have been unknown to-day. Hence the importance which attaches to methods of pulp-canal treatment and the intense activity of interest expended upon this subject in all directions.

The devices which have been tried in pulp canal treatment are innumerable, but out of the chaotic mass of crude experimentation certain fundamental principles have been evolved which are factors *sine qua non* in the process. Especially has it been established that no method can be successful that does not practically deal with the factor of bacterial infection and relate it in proper order with the associated procedures in canal treatment. It is the bacterial factor which is at the foundation of all pulp canal treatment, and successful methods are but intelligent applications of antisepsis to the several conditions to be met.

The purpose of this communication is to direct attention to two essentially different modes of dealing with pulp canals and their contents, in which the difference is so great that a discussion of their relative merits seems pertinent at this time.

One method is based upon the conception that all pulp tissue and organic matter should be removed from the root canals as a prerequisite of success, the other that the pulp-tissue may, by chemical means, be so altered as to resist infection by bacteria, and so remain inert.

The success of the first is based upon theoretically correct principles which practice has demonstrated to be true. An environment devoid of pabulum can not support bacterial life, therefore complete removal of organic matter from the canals and tubules of a tooth renders the territory uninhabitable, hence putrefactive processes with their irritative sequelæ are thus prevented. Practice has fully demonstrated the fact that an aseptic root-canal absolutely filled gives no subsequent trouble to either patient or operator. The ideal conditions for the application of this principle are, however, not always attainable. Mechanical difficulties of position, small and tortuous canals, inaccessibility, abnormal shapes of canals, etc., are practical obstacles to the complete mechanical removal of the dead pulp tissue, hence have arisen various methods based upon the second principle alluded to, of converting the pulp into an unalterable, inert mass by chemical means and allowing it to remain *in situ* as a canal filling.

There can be no doubt that this method is based upon correct principles if it can be shown that the chemical treatment of the pulp will permanently prevent its decomposition, a factor which in practice remains as yet to be proven.

There are strong analogies between the recently proposed methods of pulp mummification and the results of the earlier operations in pulp-capping by oxychloride of zinc, so far as their chemical features are concerned. Before the properties of zinc chloride were clearly understood in relation to the tissues of the dental pulp, many successful (?) cases of pulp-capping were found to be ultimately cases of successful pulp destruction. It became a notorious fact that an exposed pulp capped directly with oxychloride of zinc after a period of some disquietude eventually became comfortable and remained so until perhaps years afterward, when the operator, having occasion to renew the filling, found to his surprise an empty pulp chamber beneath his capping instead of a vital pulp, as he had expected from the fact that no acute disturbance had followed the original operation. Destruction of the pulp by oxychloride of zinc directly applied is probably the general consequence of such practice. Subsequent pericemental disturbance is usually absent because of the antiseptic properties of the capping preventing the ingress of putrefactive germs. The analogy is somewhat closer when it is remembered that the coagulum of zinc chloride and animal tissue or albuminous matter is dense and leather like, and when not plentifully saturated with moisture remains unattacked by micro-organisms indefinitely, provided no great excess of zinc chloride is present beyond the amount required to produce the coagulum. Pulps *in situ* treated with zinc oxychloride in most cases disintegrate after a certain period, leaving the canals empty, after which infection may take place.

It is probable that the various mummifying processes gaining in vogue at the present time are of more definite value and greater permanence than the results of treatment by oxychloride of zinc, but all are chemically analogous, and the differences in result are of degree rather than kind, for each and all of them depend upon a chemical tanning or coagulation of the animal tissue, converting it into a more or less unalterable compound. The practical test of experience and time alone can determine the value and utility of this class of operations as of all others.

An important feature in relation to the general introduction of mummifying methods of pulp treatment should not be lost sight of, viz., the tendency to their careless and indiscriminate use and to encourage slovenly and imperfect operations.

It is far less difficult to seal a pellet of mummifying paste into a cavity, complete the operation at a short single sitting, and collect the fee upon its completion than it is to do a thorough canal operation, and the very ease which this short cut through an essentially difficult operation to the fee for it confers is a dangerous temptation to degrade the high standard of dental work which should always be maintained. Unless it can be shown that the mummifying method is something more than a deceptive device for postponing the eventual development of apical disturbances due to ultimate failure of the protective character of chemical applications to the pulp, it should be undertaken with extreme caution until the records of its successful use have carried it beyond the purely experimental stage.

The view here presented is not intended to be condemnatory of the methods under consideration, nor of the principle upon which they are based, but merely as cautionary against a general adoption of them as methods of routine practice at this stage of their history.

The mummifying method is a direct exponent of the difficulties in the way of the older methods of canal treatment based upon the principle of complete mechanical removal of the organic canal contents. It has been claimed by some operators that no pulp canal presents insurmountable obstacles to complete mechanical cleansing. While we are willing to admit for the sake of the argument that the claim may be true for those that make it, there is no question that many cases of canal treatment occur which are beyond the ability of operators of more than average skill to successfully cleanse and fill by strictly mechanical means. Hence the necessity of some method by which the average practitioner can deal successfully with this class of cases.

Attention of the profession has been directed to another series of methods for canal treatment which have for their object the complete removal of organic matter, not by mechanical but by chemical means, and which apparently have not received the general attention to which their merits entitle them.

Reference is here made to the Callahan sulfuric acid method, the Schreier method and the sodium dioxid method. The basal principle in each of these is the same, viz., the solvent action of a chemical substance upon the canal contents whereby they may be dissolved and washed out. Sterilization is effected at the same time by the chemicals used, and the canals may be filled with a fluid of low melting point and one indestructible in the position in which it is placed.

A technique involving the principles noted leaves but little to be desired, is of general applicability and is in accord with the lines which experience has heretofore shown to be those which insure success.

We believe that further study will ultimately demonstrate the superiority of these pulp dissolving methods over the devices which permit any portion of the organic matter to remain, even when guarded by the coagulating and preserving effect of the best agents at present known for that purpose.

